# Enhancing diagnostic precision in thyroid nodule assessment: evaluating the efficacy of a novel cell preservation technique in fine-needle aspiration cytology

**DOI:** 10.3389/fendo.2024.1438063

**Published:** 2024-08-27

**Authors:** Diana-Raluca Streinu, Octavian Constantin Neagoe, Andreea Borlea, Ion Icma, Mihnea Derban, Dana Stoian

**Affiliations:** ^1^ Department of Doctoral Studies, Victor Babes University of Medicine and Pharmacy, Timisoara, Romania; ^2^ Center of Molecular Research in Nephrology and Vascular Disease, Faculty of Medicine, Victor Babes University of Medicine and Pharmacy, Timisoara, Romania; ^3^ First Surgery Clinic, “Pius Brinzeu” Clinical Emergency Hospital, Timisoara, Romania; ^4^ Second Clinic of General Surgery and Surgical Oncology, Timisoara Municipal Emergency Clinical Hospital, Timisoara, Romania; ^5^ Center for Advanced Ultrasound Evaluation, Dr. D Medical Center, Timisoara, Romania; ^6^ 2nd Department of Internal Medicine, Victor Babes University of Medicine and Pharmacy, Timisoara, Romania; ^7^ Department of Pathology, CF Clinical Hospital, Timisoara, Romania

**Keywords:** cytology, cytomatrix, thyroid nodules, fine needle aspiration, thyroidectomy, Bethesda

## Abstract

**Objectives:**

This study aimed to evaluate the effectiveness of thyroid fine needle aspiration cytology (FNAC) using a novel-cell preserving matrix called Cytomatrix in improving diagnostic accuracy for thyroid nodules.

**Materials and methods:**

Fifty patients undergoing thyroidectomy were enrolled and FNAC was performed on the excised thyroid glands, with the collected sample being placed on the Cytomatrix. The results were compared with histopathological analysis, and diagnostic performance was assessed statistically.

**Results:**

Cytomatrix demonstrated an accuracy of 96%, sensitivity of 84.61%, and specificity of 100%. Concordance between cytological and histopathological findings highlighted Cytomatrix’s potential to enhance thyroid FNAC accuracy.

**Conclusion:**

FNAC using Cytomatrix shows promise in improving diagnostic accuracy for thyroid nodules. Its application, marked by faster processing and efficient resource utilization, coupled with the preservation of cellular architecture, holds considerable potential in enhancing cytological diagnosis, thus optimizing patient management strategies.

## 1 Introduction

Thyroid nodular disease has become increasingly prevalent in clinical practice, owing to advancements in diagnostic methodologies, with a substantial majority of these nodules presenting as benign lesions (1). The continuous rise of environmental pollution (2, 3), dietary changes, radiation exposure, the use of certain medications, combined with individual genetic predisposition, represent the main contributors in the development of thyroid nodules, especially thyroid carcinoma (4–6). With advancing age, the incidence of thyroid nodules increases, showing prevalence rates ranging from 7% to 67%, and with a higher likelihood of occurrence in women (7). Thyroid nodules can be detected via self-palpation, though this depends on lesion size. More commonly, they are found incidentally during routine examinations, particularly through neck ultrasound (8). Recent years have witnessed a surge in the identification of smaller lesions, including thyroid carcinomas, which represent approximately 7-15% of all nodules (9). This rise is mainly due to advances in thyroid ultrasound technology, including elastography and contrast-enhanced ultrasound (CEUS) for assessing high-risk nodules (10–12).

Despite progress in imaging modalities for detecting suspicious thyroid lesions, distinguishing between malignant and benign nodules is a persistent challenge (13).

Fine needle aspiration cytology (FNAC) remains the gold standard in preoperative assessment of thyroid nodules, with high sensitivity and accuracy in differentiating malignant lesions, as presented in published literature, with sensitivity and specificity ratios ranging from 65% to 98% and 73% to 100%, respectively (14). FNAC of the thyroid is typically performed by specialized practitioners—endocrinologists, radiologists, or surgeons—who have undergone rigorous training (15, 16).

Experts use ultrasound guidance to precisely locate thyroid nodules. A 22-to-27-gauge needle extracts cellular material, which is then smeared on slides for cytological examination (17). Results are analyzed using the 2023 Bethesda System for Reporting Thyroid Cytopathology, which categorizes them into six groups: (i) nondiagnostic; (ii) benign; (iii) atypia of undetermined significance; (iv) follicular neoplasm; (v) suspicious for malignancy (SFM); and (vi) malignant (18).

Despite the utility of FNAC, a considerable proportion of samples yield inconclusive results, primarily due to inadequate material collection and processing, potentially resulting in interpretation errors and diagnostic uncertainty (19, 20). Immunohistochemical and molecular tests are limited with conventional smears due to cell architecture alterations. On the other hand, cell block preparation preserves cellular structure and improves diagnostic accuracy but is resource-demanding and time-consuming, leading to delays in diagnosis and management (21). It also requires more cellular material, with potential loss during preparation (22, 23).

The Cytomatrix (developed by UCS Diagnostic Srl & Campus Bio-Medico University of Rome, Rome, Italy) represents a cutting-edge advancement in cell preservation techniques tailored specifically for capturing and preserving the architecture of thyroid cells obtained after FNAC within its 3D synthetic matrix (24). This unique feature enables the obtained cytology to closely mimic the patterns observed in histological sections, requiring minimal intervention from the technician post-sample collection, and thereby substantially enhancing the accuracy of diagnosis.

Moreover, the preserved matrix can be stored indefinitely, offering the flexibility to perform additional diagnostic tests such as immunohistochemistry (IHC), fluorescence *in situ* hybridization (FISH), and molecular biology assays. Additionally, it presents the advantage of requiring a smaller volume of cell aspirate compared to traditional smears or cell blocks (24). This not only streamlines the sample collection process but also preserves valuable cellular material for further analyses, ultimately contributing to more comprehensive diagnostic evaluations.

The primary objective of this study was to assess the capability of the novel Cytomatrix in delivering accurate and precise diagnosis following FNAC of the suspicious thyroid nodules, by comparing the Cytomatrix cytological findings with the histopathological results of the excised thyroid glands, aiming to minimize the occurrence of inconclusive results or misdiagnoses, commonly associated with traditional cytology smears. Emphasis was placed on prioritizing an approach that is resource-efficient and time-effective.

## 2 Materials and methods

This prospective investigation centered on patients admitted to the First Surgical Department of Timisoara Emergency County Hospital, between March 2023 and March 2024, specifically those scheduled for thyroidectomy. Selection for surgery was based on suspicious ultrasound findings, the presence of compressive symptoms, and additional factors such as elevated calcitonin levels or a clinical diagnosis of Graves’ disease in patients who did not respond to antithyroid medication. Fine needle aspiration cytology (FNAC) was performed on the excised thyroid gland of each patient enrolled. The selection criteria for performing FNAC on the specimens was established based on preoperative ultrasound evaluations, characterized by at least one suspicious nodule scoring 4 or higher on the European Thyroid Imaging and Reporting Data System (EU-TIRADS) (25). Preoperative FNAC was not conducted. Subsequently, fifty patients meeting these criteria were enrolled.

Adhering to the principles outlined in the Declaration of Helsinki, informed consent was obtained from all participants.

**Figure 1:**
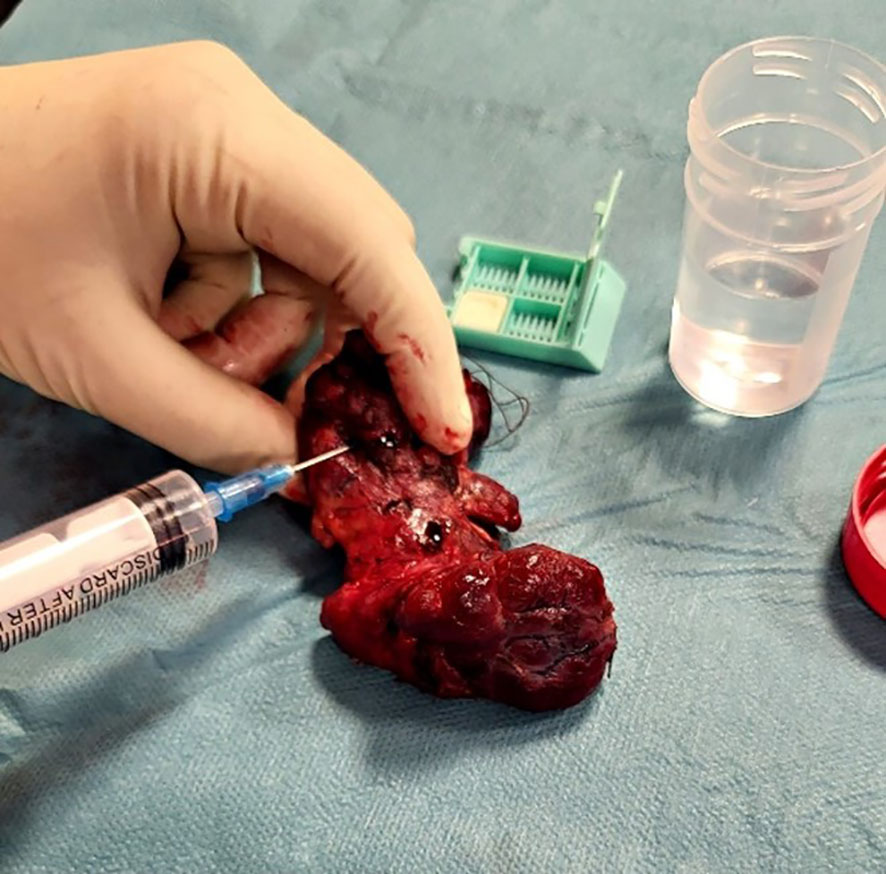
FNA technique on the excised thyroid gland.

Following the surgical excision of the thyroid gland, the surgeon promptly conducted fine needle aspiration on the thyroid’s most suspicious nodule, as determined by the preoperative ultrasound examination, prior to its immersion in formalin for subsequent histopathological analysis. The excised thyroid was placed on a sterile surface. Subsequently, the surgeon located the preoperative ultrasound-identified suspicious nodule through palpation. A 23-gauge needle attached to a 10ml syringe was used for sample collection (as shown in **Figure 1**). Combined capillary and aspiration techniques were used.

**Figure 2:**
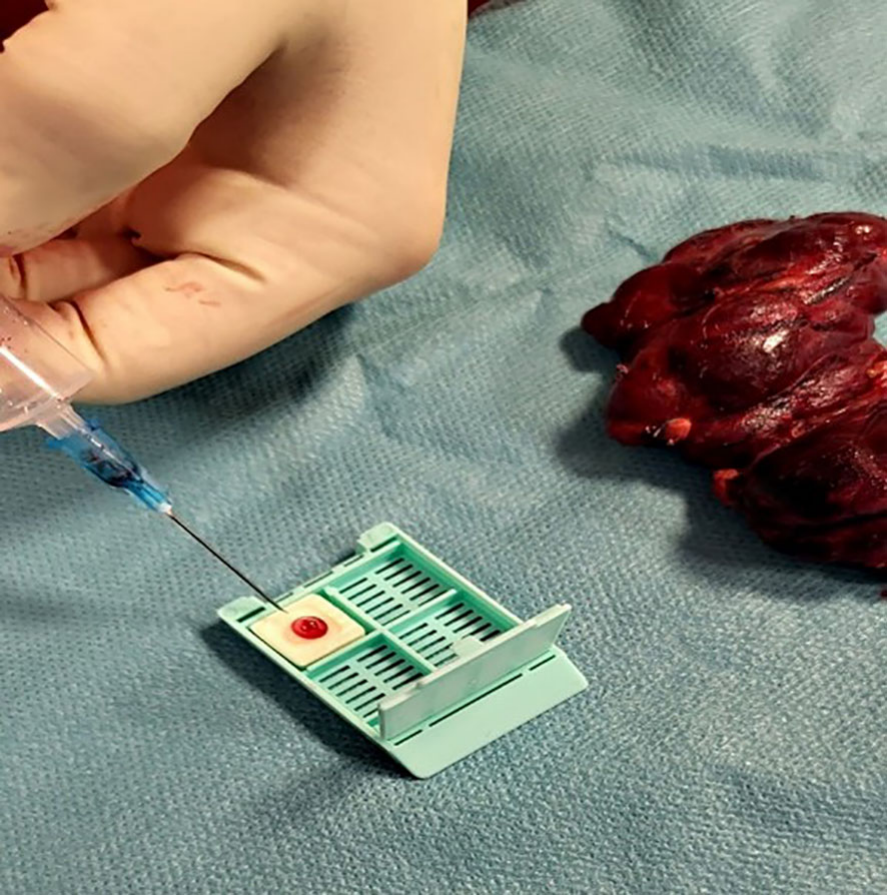
Placement of obtained material onto the Cytomatrix.

After the fine needle aspiration process, the collected sample (1-2 drops) was placed onto the Cytomatrix sponge (as shown in **Figure 2**) and immersed in 30ml formalin for at least 12 hours.

After fixation in formalin, the Cytomatrix underwent permanent embedding in paraffin and was subsequently sectioned using a microtome to generate multiple 10-micron slices, which were then subjected to standard histological hematoxylin and eosin (HE) staining procedures. The excised thyroid gland underwent histopathological examination at the pathology department of the hospital using standard diagnostic protocols. The results were interpreted in accordance with the 2022 World Health Organization (WHO) histologic classification of thyroid neoplasms (26). To maintain a blind approach, the cytological analysis of the Cytomatrix sections was performed by a different pathologist than the one who conducted the histopathological examination of the excised specimens. Furthermore, the histopathological evaluation was carried out at a separate center to ensure unbiased results.

Because of the non-standardized nature of Cytomatrix, the cytological results were stratified into categories mirroring those of the 2023 Bethesda System, encompassing benign, equivocal, suspicious for malignancy, and malignant. This categorization was employed to establish a comprehensive diagnostic framework consistent with the principles of the 2023 Bethesda System. Cases in which acellular samples were acquired during FNAC, resulting in the production of non-diagnostic Cytomatrix outcomes akin to the Bethesda I category, were excluded from the analysis.

Ultimately, the cytology results were compared with the histopathological findings.

Fifty patients were included in the study, comprising individuals who underwent both histopathological and cytological examinations. The demographic characteristics, including age and sex of each participant, were recorded.

Cytological assessments were conducted on the Cytomatrix block. The results were categorized according to the classification scheme designed to parallel the 2023 Bethesda System.

Histopathological examinations were conducted on the excised thyroid gland in accordance with the 2022 World Health Organization (WHO) histologic classification of thyroid neoplasms, to confirm the presence of malignant or benign lesions. The number of patients where malignancy was present was recorded, along with the specific subtypes identified, and the number of patients with benign lesions, along with their subtypes were also documented.

The comparison between the Cytomatrix cytology results and histopathological findings are illustrated through descriptive statistics, including graphical representations. These representations encompass the prevalence of identified malignancies and their subtypes, benign lesions, and their subtypes, as well as demographic variables within the cohort of fifty patients. Statistical analysis was carried out using MedCalc Statistical Software version 20.111 (MedCalc Software Ltd., Ostend, Belgium).

## 3 Results

Fifty patients underwent total thyroidectomy in this study, with ages ranging from 21 to 74 years old. 7 were male and 43 were female.

Only nodules with a minimum preoperative ultrasonography (US) risk assessment of 4 (according to the US EU-TIRADS classification) were subjected to FNAC and were distributed as follows: 40 nodules classified as TIRADS 4 and 10 nodules classified as TIRADS 5.

In the cohort of fifty patients analyzed using the Cytomatrix methodology, the results were classified according to the classification employed to mirror the Bethesda system. The analysis yielded 37 cases classified in the benign category, 2 as equivocal, 9 as suspicious for malignancy, and 2 as malignant.

The Cytomatrix cytological results were validated in accordance with the final histological examination that uncovered 13 malignancies within the fifty-patient cohort. This comprised 9 instances of papillary carcinoma and 2 instances of follicular variant of papillary carcinoma, each in alignment with its corresponding Cytomatrix diagnosis falling under the suspicious for malignancy or malignant category. The two cases initially categorized as equivocal risk on the Cytomatrix were subsequently identified as medullary carcinoma and follicular variant of papillary carcinoma upon histopathological examination.

Furthermore, the final histological examination classified the remaining 37 cases as benign lesions, mirroring the results of the Cytomatrix cytological analysis, which had categorized each of the corresponding 37 cases into the benign category. Their distribution was as follows: 28 cases of benign nodular goiter, 7 cases of chronic thyroiditis, and 2 cases of Graves disease. Although these two cases were initially diagnosed clinically preoperatively, both Cytomatrix and histopathological examinations detected microscopic features often associated with Graves’ disease, such as hyperplasia and hypertrophy of the follicular cells, as well as lymphocytic infiltration. These findings further reinforced the diagnosis.

Out of the total fifty patients, Cytomatrix identified 11 malignancies (True Positives) 37 benign lesions (True Negatives), and 2 equivocal lesions (False Negatives). Histopathological examination identified 13 malignancies, and 37 benign lesions.

After the statistical analysis of the obtained data, the Cytomatrix cytological analysis exhibited an accuracy of 96%. Additionally, it revealed a sensitivity of 84.61% and a specificity of 100%. Furthermore, the positive predictive value (PPV) of the Cytomatrix was determined to be 100%. The two cases categorized as equivocal by the Cytomatrix were labeled as false negatives, contributing to a discordance rate of 15.39% between Cytomatrix and the histopathological exam.

**Figure 3:**
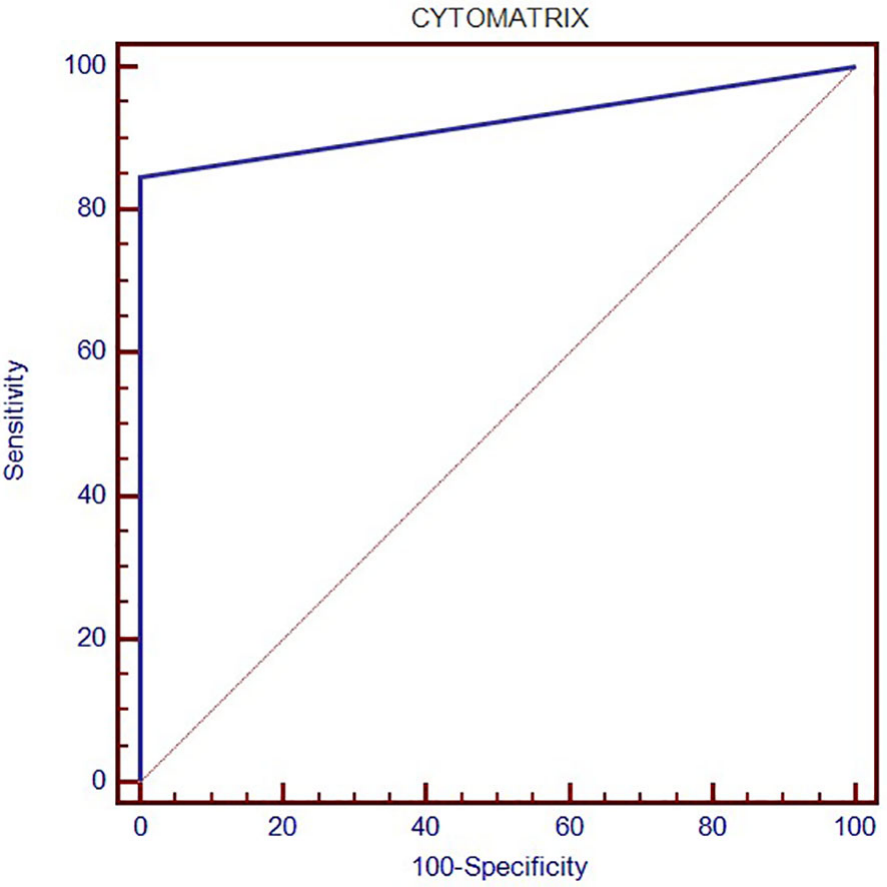
Receiver Operating Characteristic (ROC) curve, which illustrates the diagnostic performance of the Cytomatrix technology, showing both high sensitivity and specificity.

A graphical representation of the Cytomatrix performance is shown in **Figure 3**.

**Figure 4:**
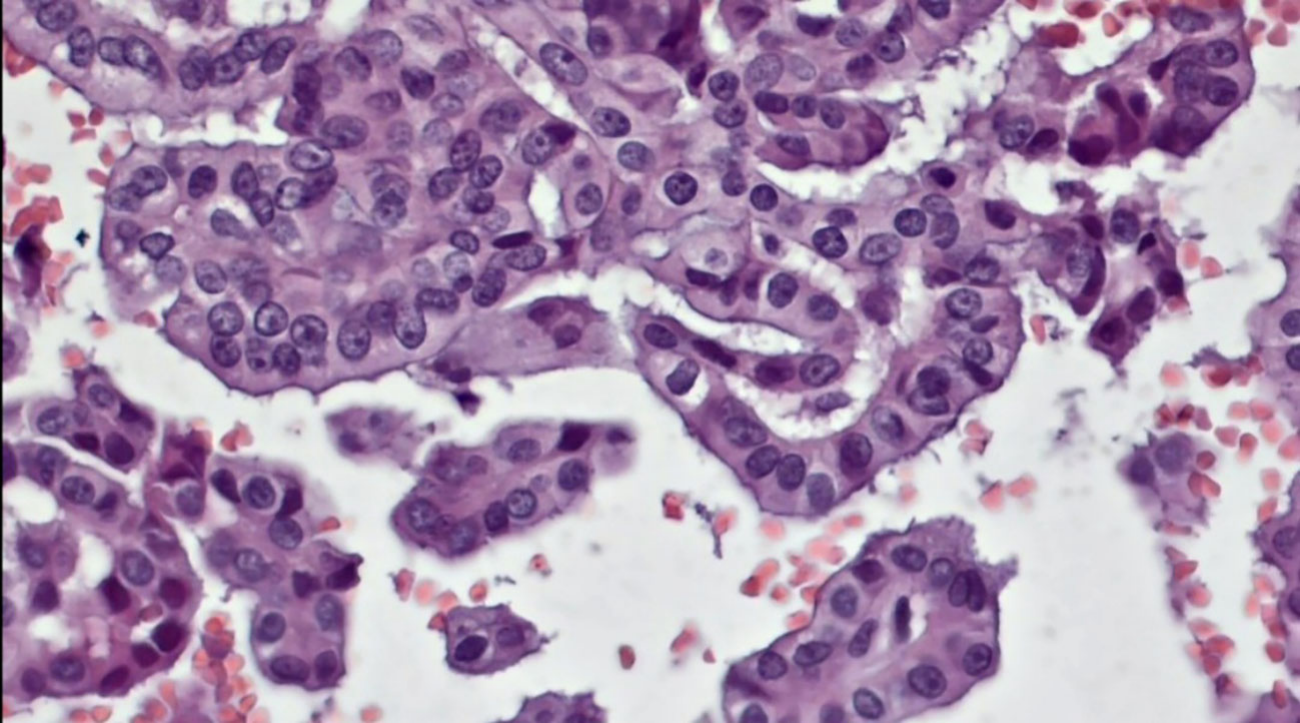
Slides cut from the paraffin-embedded Cytomatrix showcasing papillary carcinoma – magnification 40x.

**Figure 5:**
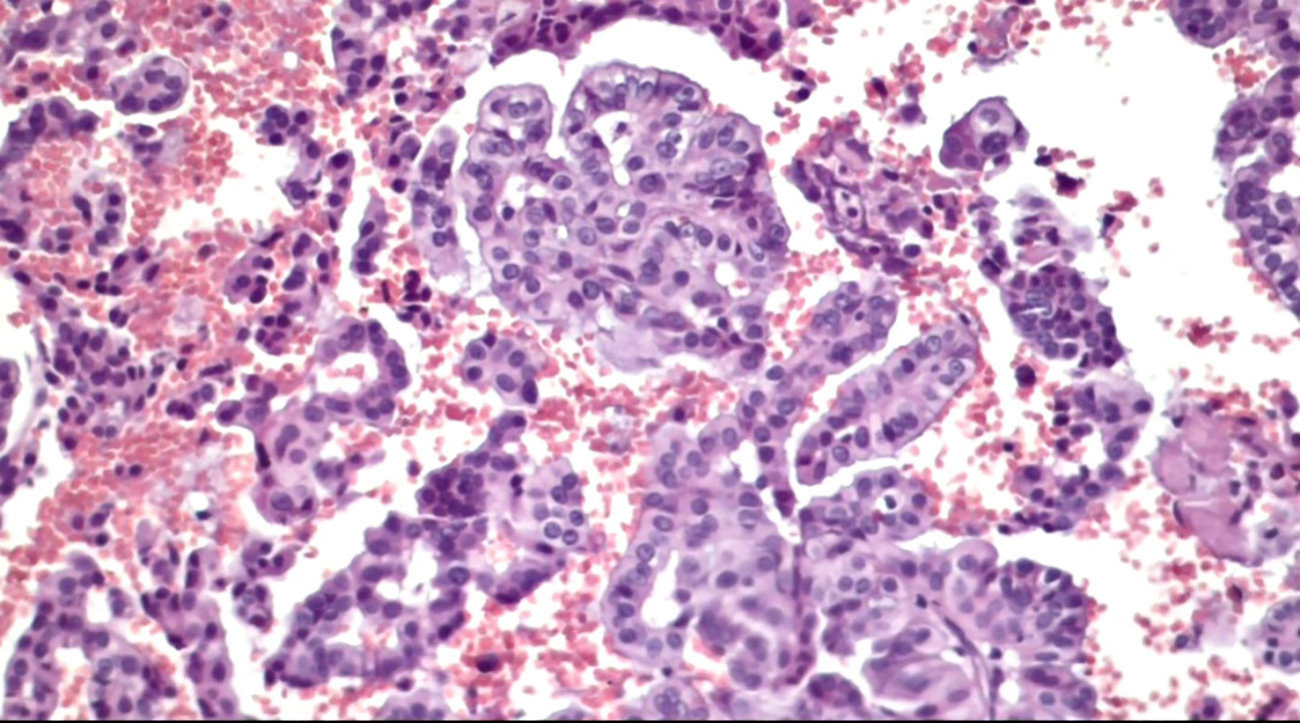
Slides cut from the paraffin-embedded Cytomatrix showcasing papillary carcinoma – magnification 20x.

**Figure 6:**
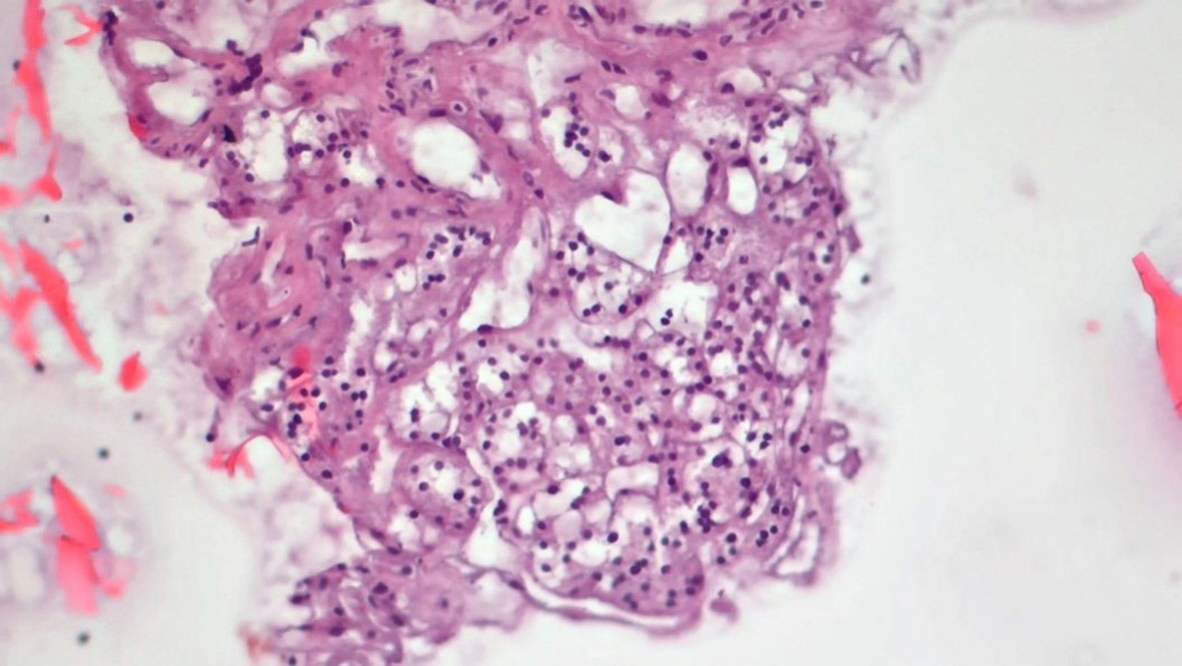
Slides cut from the paraffin-embedded Cytomatrix showcasing benign nodular disease – magnification 10x.

**Figure 7:**
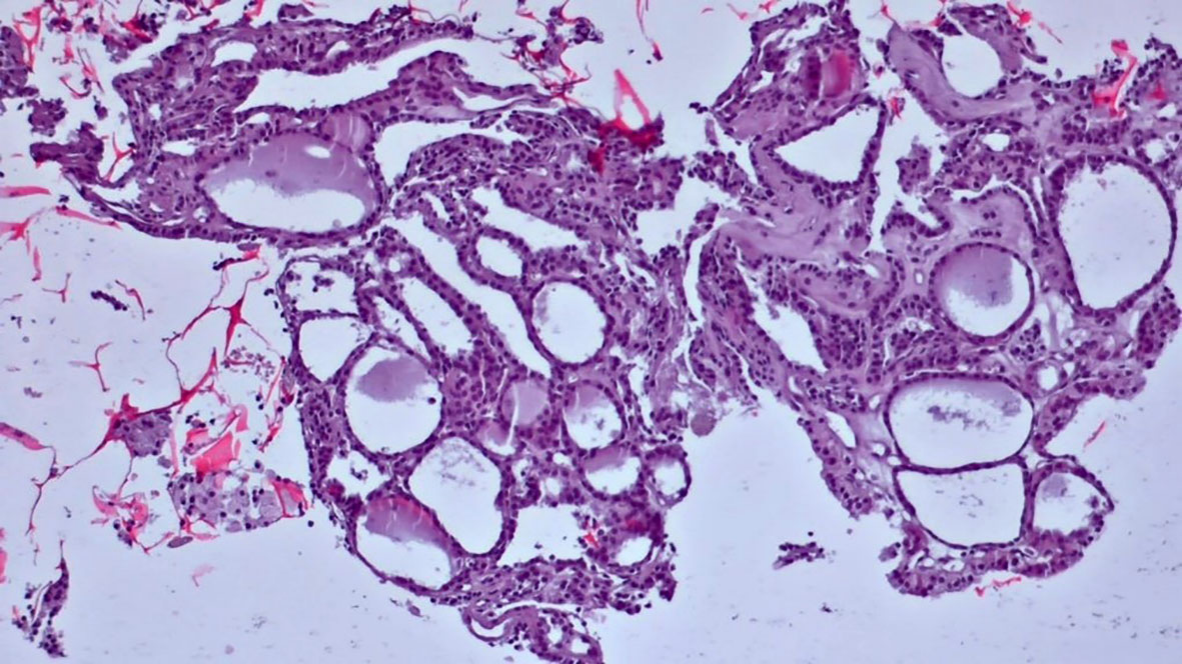
Slides cut from the paraffin-embedded Cytomatrix showcasing benign nodular disease – magnification 10x.

Obtained results and demographic data are represented in **Table 1** (Abbreviations: SFM – Suspicious For Malignancy). Microscopic images depicting various instances of Cytomatrix findings from the fifty-patient cohort are displayed in **Figure 4** showcasing papillary carcinoma with 40x magnification, **Figure 5** showcasing papillary carcinoma with 20x magnification, and **Figures 6**, **7** showcasing benign nodular goiter with 10x magnification.

**Table 1:** Representation of the concordance between the Cytomatrix cytological results and histopathological results among the fifty-patient cohort.

Row Labels	Instances
**BETHESDA II equivalent - BENIGN**	**37**
** Chronic Thyroiditis**	**7**
Female	7
** Benign Nodular Goiter**	**28**
Male	5
Female	23
** Graves Disease**	**2**
Female	2
**BETHESDA III equivalent - EQUIVOCAL**	**2**
** Medullary Carcinoma**	**1**
Female	1
** Follicular Variant of Papillary Carcinoma**	**1**
Female	1
**BETHESDA V equivalent - SFM**	**9**
** Papillary Carcinoma**	**7**
Male	1
Female	6
** Follicular Variant of Papillary Carcinoma**	**2**
Male	1
Female	1
**BETHESDA VI equivalent - MALIGNANT**	**2**
** Papillary Carcinoma**	**2**
Female	2
**Grand Total**	**50**

SFM, Suspicious for Malignancy.

The words in bold represent the Bethesda categories and specific diagnoses within each category. These are highlighted to distinguish the classification and diagnosis of thyroid lesions, making it easier to differentiate between the various diagnostic outcomes.

## 4 Discussions

The objective of this investigation was to evaluate the effectiveness of a novel cell-preserving matrix, known as Cytomatrix, in thyroid FNAC. By conducting a comparative assessment of the cytological outcomes obtained through Cytomatrix with the subsequent histological findings from the excised thyroid specimens of the same cohort of fifty patients, a valid concordance between the two diagnostic methods was established.

Cytomatrix exhibited encouraging outcomes, with a sensitivity of 84.61% and a specificity of 100%, reflecting its enhanced capacity to detect malignant cases among all true positives and its capacity in distinguishing benign lesions among the 37 true negative cases, respectively. Moreover, the positive predictive value (PPV) of Cytomatrix was established at 100%, underscoring its reliability in forecasting malignancies among the cases identified as true positives. The accuracy of 96% is comparable to values found in published data regarding the accuracy of conventional smears in FNAC of the thyroid (27), further reinforcing the potential and efficacy of Cytomatrix in thyroid FNAC, providing a comprehensive evaluation of its diagnostic potential.

These findings show promise, especially when compared with published results obtained through conventional smear techniques. While certain studies have indicated lower sensitivity, specificity, and PPV with conventional smears in thyroid FNAC (28), others have reported outcomes like those observed in this study (29). Nonetheless, these findings emphasize the potential of Cytomatrix as a valuable tool in thyroid fine-needle aspiration cytology (FNAC).Two cases of equivocal lesions (equivalent to the Bethesda III category of indeterminate lesions) were identified on the Cytomatrix from the cohort of fifty patients, both of which were subsequently confirmed as malignant upon histological examination. This observation is consistent with existing literature suggesting a higher likelihood of malignancy confirmation upon histological evaluation for lesions categorized as Bethesda III (30). As only two cases in this study were categorized as equivocal lesions (equivalent to Bethesda III indeterminate lesions), it raises questions regarding the potential efficacy of Cytomatrix in reducing the occurrence of indeterminate lesions, particularly within the Bethesda III category. It prompts an inquiry into whether the use of conventional smears would have yielded more cases of indeterminate lesions (Bethesda III) within the fifty-patient cohort, or if the use of Cytomatrix genuinely contributed to minimizing such outcomes. Indeterminate lesions, specifically those falling into the Bethesda III category, are well-known for presenting a diagnostic challenge (31). However, Cytomatrix offers the possibility to perform additional ancillary tests, thereby enhancing diagnostic accuracy (24, 32).

Traditional smears remain a reliable, cost-effective, and straightforward method in thyroid (FNAC). Nonetheless, a notable proportion of cases analyzed using traditional smears yield inconclusive results or misdiagnoses, largely attributed to operator-dependent factors such as inadequate or insufficient material collection during the FNAC procedure (33, 34). This underscores the necessity for repeated FNAC or multiple passes to obtain an adequate sample, resulting in a time-consuming, cost-ineffective, and burdensome process for the patient (35, 36). In contrast, Cytomatrix requires a smaller volume of sampled material due to its distinctive 3D cell-preserving structure. With Cytomatrix, cells retain their original architecture instead of being smeared, allowing for a more efficient use of the sampled material during analysis. In each of the fifty cases presented in this study, 1-2 drops of FNAC sample placed on the Cytomatrix sponge, along with a single needle pass, proved adequate for achieving a reliable cytological diagnosis.

Both capillary and aspirate techniques were used in the material collection process during fine needle aspiration. The capillary method, regarded as a non-aspiration technique, is employed for its ability to minimize hemorrhagic material collection, as blood can often compromise sample adequacy. Although fine needle capillary sampling (FNCS) yields higher-quality material, fine needle aspiration cytology (FNAC) offers superior quantitative sampling. Each technique presents distinct advantages and limitations. A hybrid approach, incorporating both methods might yield superior fine needle cytology samples and enhance diagnostic accuracy (37, 38).

As indicated, non-diagnostic (Bethesda I equivalent) samples were omitted from the scope of this study.

Similar to conventional smears, Cytomatrix also carries the potential for generating non-diagnostic or inconclusive outcomes, largely due to insufficient cellular material. However, this risk is notably reduced by its distinctive capability to preserve collected material more effectively (24). Consequently, even with a lower quantity of cells, there is a higher likelihood of maintaining the unaltered quality of the sample. This characteristic distinguishes it from traditional smears, where the smearing process, along with subsequent fixation and staining techniques, poses a heightened risk of artifacts, cell overlap, morphological alterations of the sampled material, and potential cell loss (39, 40).

Another well-established method in thyroid cytology is represented by the use of cell blocks.

This technique has demonstrated its efficacy in diagnosis, primarily attributed to the superior preservation of cell architecture within the block, the same advantage Cytomatrix offers. Furthermore, the versatility of cell blocks extends beyond traditional cytological analysis offered by conventional smears, allowing for additional optimized ancillary tests such as molecular analysis, immunohistochemistry (ICH) or FISH (Fluorescence *In Situ* Hybridization). This multifaceted approach enhances the diagnostic capabilities, providing a more intricate analysis of the sampled material (41, 42).

The cell block technique encompasses various preparation methods, such as thromboplastin-plasma clot method, fibrin clot method, or cell blocks from liquid-based cytology. Each of these methods presents its unique approach (21, 43–45).

However, the creation of a single cell block demands additional cellular aspirate, as some of the collected material may be lost, depending on the type of processing technique (22, 46).

Therefore, it’s noteworthy that regardless of the specific cell block technique employed, most of these methods share a common trait — they tend to be time-consuming and necessitate additional specialized equipment for optimal execution, and sometimes offer no added benefit to the final interpretation, resulting in delays in diagnosis and patient management (21, 47).

Cytomatrix, however, effectively addressed these limitations in this study, as there was no longer need for additional specialized equipment. Its distinctive design enabled the operator to directly deposit the collected material from FNAC of the thyroid, typically comprising 1-2 drops, onto the preserving sponge of the Cytomatrix complex, eliminating the requirement for additional processing. Following immersion in formalin, the Cytomatrix was embedded in paraffin and cut using a microtome, thus greatly simplifying the procedure for the final analysis.

One of the limitations encountered was insufficient cells on the matrix. These cases, as presented, were excluded from the study. However, it is important to emphasize that this limitation solely stemmed from the operator’s sampling technique. There were no instances of cell loss or alterations attributable to the Cytomatrix itself or its processing technique, as observed in the processing of conventional smears or various cell block preparation methods (22, 39).

Another advantage of the Cytomatrix is its capacity to generate multiple sections from the preserved paraffin block, facilitating long-term storage and future analyses. This sets it apart from conventional cytological smears, which are generally limited to a single use (25, 48). The Cytomatrix blocks obtained from the fifty-patient lot can be further used to perform additional ancillary tests, such as IHC, FISH and molecular biology, that are not otherwise optimized to be performed on conventional smears.

The integration of ancillary tests in thyroid cytology enhances diagnostic precision, enables personalized treatment approaches, and provides valuable insights into the molecular characteristics of thyroid lesions. These advanced techniques contribute to a more nuanced understanding of thyroid pathology, particularly in navigating the diagnostic complexities associated with well-known indeterminate lesions (Bethesda III), assisting clinicians in making informed decisions regarding patient management (49, 50).

In the current study, incorporating ancillary tests was deliberately avoided, emphasizing the commitment to assess the potential of Cytomatrix in thyroid FNAC while minimizing resource utilization. The assessment of its diagnostic efficacy, compared to conventional smears, relied solely on routine cytological analysis. This strategic approach aimed to emphasize the efficiency and cost-effectiveness of the Cytomatrix technique, even in the absence of supplementary ancillary testing. It underscores the potential of Cytomatrix in achieving precise diagnoses with minimal resource requirements, showcasing its effectiveness in routine practice.

Nevertheless, it is important to highlight that some studies opted to incorporate ancillary test in their evaluation using Cytomatrix, resulting in successful outcomes (51, 52). This highlights the versatility and effectiveness of the Cytomatrix, particularly when researchers choose to explore its full potential through these additional analyses.

The pathologist’s perspective further showcases the efficacy of the Cytomatrix. When presented with matrices containing adequate material for analysis, the pathologist remarked that the experience was akin to examining a histology slide rather than traditional cytology. This observation not only emphasizes the matrix’s effectiveness but also underscores its potential to elevate the diagnostic process to a level reminiscent of histopathological assessments.

The cytological characteristics of thyroid lesions play a crucial role in diagnostic accuracy and subsequent patient management. To evaluate the effectiveness of different cytological diagnostic methods in thyroid FNAC, a comparative analysis of Cytomatrix, conventional smears, and histological analysis was conducted. **Table 2** presents a comprehensive comparison of these methods based on various parameters such as cellular architecture, nuclear traits, presence of artifacts, and ancillary testing capabilities (24, 53, 54). While these traits are primarily based on established literature, some of them are reinforced by the findings from this study. It is meant to highlight that Cytomatrix has the potential to be a significant advancement in thyroid fine-needle aspiration cytology (FNAC) compared to conventional smears. The close resemblance between Cytomatrix cytological analysis and the histological examination underscores its role in bridging the gap between cytology and histology. This positions Cytomatrix as a promising advancement in thyroid FNAC, offering the potential for enhanced diagnostic accuracy and supporting more precise patient management strategies.

**Table 2:** Comparison between Cytomatrix, conventional smears and histopathological exam in thyroid FNAC.

Exam Type Traits	Cytomatrix	Conventional Smears	Histopathological Exam
**Cellular Architecture**	**Preserved** Well-preserved cellular arrangements (follicles, micro follicles, papillae)	**Limited** Poorly preserved cellular arrangements (follicles, micro follicles, and papillae)	**Preserved** Well-preserved cellular arrangements
**Nuclear Characteristics**	**Preserved** view of clearings, grooves, pseudo inclusions, and overlapping	**Limited** view of clearings, grooves, pseudo inclusions, and overlapping	**Preserved** view of clearings, grooves, pseudo inclusions, and overlapping
**Artifacts**	**Limited**	**Common** (fibrin inclusion/obscuring blood due to smearing process)	**Limited**
**Ancillary Testing**	**Suitable**	**Limited**	**Suitable**

The bolded terms "Cellular Architecture," "Nuclear Characteristics," "Artifacts," and "Ancillary Testing" represent specific traits being evaluated across the three different exam types. The words "Preserved," "Limited," and "Suitable" are also bolded to highlight how each trait is assessed for each exam type.

One significant limitation of this study is that FNAC was performed on excised thyroid glands, rather than preoperatively, directly on the patients. This approach was chosen because the study is a preliminary evaluation of the relatively new Cytomatrix technology to determine whether it can be used for cytological evaluation of thyroid lesions with minimal processing after FNAC sample collection. Another limitation encountered was the inadequacy of the sampled material, specifically the insufficient number of cells on certain Cytomatrixes, which led to their exclusion from the analysis. Future research will try to address these limitations by performing preoperative FNAC on patients *in vivo*, using Cytomatrix. The insights gained from this study will help refine the technology and validate its effectiveness for direct clinical application.

## 5 Conclusions

This study shows that Cytomatrix is a promising advancement in thyroid FNAC, effectively preserving cell architecture and offering high diagnostic accuracy. With a sensitivity of 84.61% and a specificity of 100%, Cytomatrix proved reliable in identifying malignant lesions, showing a potential improvement over conventional smears. The positive predictive value of 100% further supports its efficacy in detecting malignancies. Although the Cytomatrix identified two cases as equivocal, which were later confirmed as malignant, its overall performance aligns well with histopathological results, showcasing an accuracy of 96%.

The study highlights Cytomatrix’s capability to maintain cellular architecture and support additional testing, with minimal sample material required. This approach not only streamlines the diagnostic process but has the potential to reduce the need for repeated FNACs. The integration of Cytomatrix into routine practice could significantly enhance diagnostic precision, reduce resource use, and improve patient management by minimizing inconclusive results. Future research will focus on validating Cytomatrix in preoperative, *in vivo* FNAC scenarios to further refine its clinical application and address the limitations observed in this pilot study.

## Data Availability

The original contributions presented in the study are included in the article/supplementary material. Further inquiries can be directed to the corresponding author.

## References

[1] Dean DS Gharib H. Epidemiology of thyroid nodules. Best Pract Res Clin Endocrinol Metab. (2008) 22:901–11. doi: 10.1016/j.beem.2008.09.019 19041821

[2] Mustață D-M Ionel I Popa R-M Dughir C Bisorca D. A study on particulate matter from an area with high traffic intensity. Appl Sci. (2023) 13:8824. doi: 10.3390/app13158824

[3] Popa R-M Mustață D-M Ionel I Balogh R-M. Metal element traces sampled from peri-urban road verge particulate matter. Appl Sci. (2023) 13:11649. doi: 10.3390/app132111649

[4] Zhang Y Wang K Qin W Jin C Song Y Jia P. Six air pollutants associated with increased risk of thyroid nodules: A study of 4.9 million chinese adults. Front Endocrinol (Lausanne). (2021) 12:753607. doi: 10.3389/fendo.2021.753607 34966357 PMC8710776

[5] Kermoison G Draganescu C. Role of dietary and environmental factors on thyroid cancer in Romania: A brief review. Diagnostics. (2022) 12:1959. doi: 10.3390/diagnostics1208195 36010309 PMC9406885

[6] Feier CVI Vonica RC Faur AM Streinu DR Muntean C. Assessment of thyroid carcinogenic risk and safety profile of GLP1-RA semaglutide (Ozempic) therapy for diabetes mellitus and obesity: A systematic literature review. Int J Mol Sci. (2024) 25:4346. doi: 10.3390/ijms25084346 38673931 PMC11050669

[7] Tran NQ Le BH Hoang CK Nguyen HT Thai TT. Prevalence of thyroid nodules and associated clinical characteristics: Findings from a large sample of people undergoing health checkups at a university hospital in Vietnam. Risk Manag Healthc Policy. (2023) 16:899–907. doi: 10.2147/RMHP.S410964 37220482 PMC10200104

[8] Wiest PW Hartshorne MF Inskip PD Crooks LA Vela BS Telepak RJ. Thyroid palpation versus high-resolution thyroid ultrasonography in the detection of nodules. J Ultrasound Med. (1998) 17:487–96. doi: 10.7863/jum.1998.17.8.487 9697951

[9] Pellegriti Frasca F Regalbuto C Squatrito S Vigneri R. Worldwide increasing incidence of thyroid cancer: update on epidemiology and risk factors. J Cancer Epidemiol. (2013) 2013:965212. doi: 10.1155/2013/965212 23737785 PMC3664492

[10] Stoian D Bogdan T Craina M Craciunescu M Timar R Schiller A. Elastography: A new ultrasound technique in nodular thyroid pathology. In: Thyroid Cancer - Advances in Diagnosis and Therapy. Croatia: InTech (2016). doi: 10.5772/64374

[11] Sorrenti S Dolcetti V Fresilli D Del Gaudio G Pacini P Huang P. The role of CEUS in the evaluation of thyroid cancer: From diagnosis to local staging. J Clin Med. (2021) 10:4559. doi: 10.3390/jcm10194559 34640574 PMC8509399

[12] Stoian D Borlea A Moisa-Luca L Paul C. Multiparametric ultrasound-based assessment of overt hyperthyroid diffuse thyroid disease. Front Endocrinol (Lausanne). (2023) 14:1300447. doi: 10.3389/fendo.2023.1300447 38179308 PMC10764279

[13] Chaudhary V Bano S. Thyroid ultrasound. Indian J Endocrinol Metab. (2013) 17:219–27. doi: 10.4103/2230-8210.109667 PMC3683194 23776892

[14] Chandio A Shaikh Z Chandio K Naqvi SM Naqvi SA. Accuracy of FNAC in diagnosis of thyroid gland diseases. Nurs Palliat Care. (2018) 3. doi: 10.15761/NPC.1000183

[15] Feingold KR Anawalt B Blackman MR Boyce A Chrousos G Corpas E. Fine-needle Aspiration of the Thyroid Gland. Feingold KR Anawalt B Blackman MR, editors. South Dartmouth (MA: MDText.com, Inc (2023).

[16] Wang J Zhu Y Song Y Xu G Yu H Wang T. Determining whether surgeons perform thyroid fine-needle aspiration as well as radiologists: an analysis of the adequacy and efficiency of ultrasound-guided fine-needle aspiration performed by newly trained head and neck surgeons and radiologists. Gland Surg. (2020) 9:711–20. doi: 10.21037/gs.2020.03.34 PMC7347813 32775261

[17] Mathew S Murphy A Chieng R. Ultrasound-guided FNA of the thyroid . Radiopaedia.org (Accessed 19 Jan 2024).

[18] Ali SZ Baloch ZW Cochand-Priollet B Schmitt FC Vielh P VanderLaan PA. The 2023 bethesda system for reporting thyroid cytopathology. Thyroid. (2023) 33:1039–44. doi: 10.1089/thy.2023.0141 37427847

[19] Al Maqbali T Tedla M Weickert MO Mehanna H. Malignancy risk analysis in patients with inadequate fine needle aspiration cytology (FNAC) of the thyroid. PLoS One. (2012) 7:e49078. doi: 10.1371/journal.pone.0049078 23185295 PMC3501514

[20] Bhartiya R Mallik M Kumari N Prasad BN. Evaluation of thyroid lesions by fine-needle aspiration cytology based on Bethesda system for reporting thyroid cytopathology classification among the population of South Bihar. Indian J Med Paediatr Oncol. (2016) 37:265–70. doi: 10.4103/0971-5851.195742 PMC5234164 28144094

[21] Saharti S. The diagnostic value of add-on thyroid cell block in the evaluation of thyroid lesions. CytoJournal. (2023) 20:3. doi: 10.25259/Cytojournal_9_2022 36895260 PMC9990845

[22] La Fortune KA Randolph ML Wu HH Cramer HM. Improvements in cell block processing: The Cell-Gel method. Cancer Cytopathology. (2017) 125:267–76. doi: 10.1002/cncy.21814 28140513

[23] Saharti S. Contemporary art of cell-block preparation: overview. Cytojournal. (2024) 21:5. doi: 10.25259/Cytojournal_56_2023 38343761 PMC10858773

[24] Cytomatrix. Available online at: https://cytomatrix.it/ (Accessed April 15 2024).

[25] Smith D Campos A Knipe H. European Thyroid Association TIRADS . Radiopaedia.org (Accessed 11 Mar 2024).

[26] Jung CK Bychkov A Kakudo K. Update from the 2022 world health organization classification of thyroid tumors: A standardized diagnostic approach. Endocrinol Metab (Seoul). (2022) 37:703–18. doi: 10.3803/EnM.2022.1553 PMC9633223 36193717

[27] Cramer H. Fine-needle aspiration cytology of the thyroid. Cancer. (2000) 90:325–9. doi: 10.1002/1097-0142(20001225)90:6<>1.0.CO;2-Z 11156514

[28] Erkinuresin T Demirci H. Diagnostic accuracy of fine needle aspiration cytology of thyroid nodules. Diagnosis (Berl). (2020) 7:61–6. doi: 10.1515/dx-2019-0039 32037778

[29] Machała E Sopiński J Iavorska I Kołomecki K. Correlation of fine needle aspiration cytology of thyroid gland with histopathological results. Pol Przegl Chir. (2018) 90:1–5. doi: 10.5604/01.3001.0012.4712 30652691

[30] Mulita F Plachouri MK Liolis E Vailas M Panagopoulos K Maroulis I. Patient outcomes following surgical management of thyroid nodules classified as Bethesda category III (AUS/FLUS). Endokrynol Pol. (2021) 72:143–4. doi: 10.5603/EP.a2021.0018 33749812

[31] Bongiovanni M Bellevicine C Troncone G Sykiotis GP. Approach to cytological indeterminate thyroid nodules. Gland Surg. (2019) 8:S98–S104. doi: 10.21037/gs.2018.12.06 31475096 PMC6694028

[32] Rossi ED Larocca LM Pantanowitz L. Ancillary molecular testing of indeterminate thyroid nodules. Cancer Cytopathol. (2018) 126 Suppl 8:654–71. doi: 10.1002/cncy.22012 30156775

[33] Zhu Y Song Y Xu G Fan Z Ren W. Causes of misdiagnoses by thyroid fine-needle aspiration cytology (FNAC): our experience and a systematic review. Diagn Pathol. (2020) 15:1. doi: 10.1186/s13000-019-0924-z 31900180 PMC6942345

[34] Sujatha R Gayathri J Jayaprakash HT. Thyroid FNAC: practice and pitfalls. IJPO. (2017) 4:203–6. doi: 10.18231/2394-6792.2017.0042

[35] Chow LS Gharib H Goellner JR van Heerden JA. Nondiagnostic thyroid fine-needle aspiration cytology: management dilemmas. Thyroid. (2001) 11:1147–51. doi: 10.1089/10507250152740993 12186502

[36] Espinosa De Ycaza AE Lowe KM Dean DS Castro MR Fatourechi V Ryder M. Risk of Malignancy in thyroid nodules with non-diagnostic fine-needle aspiration: A retrospective cohort study. Thyroid. (2016) 26:1598–604. doi: 10.1089/thy.2016.0096 PMC5105349 27549368

[37] Pinki P Alok D Ranjan A Nanak Chand M. Fine needle aspiration cytology versus fine needle capillary sampling in cytological diagnosis of thyroid lesions. Iran J Pathol. (2015) 10:47–53. 26516325 PMC4539790

[38] Maurya AK Mehta A Mani NS Nijhawan VS Batra R. Comparison of aspiration vs non-aspiration techniques in fine-needle cytology of thyroid lesions. J Cytol. (2010) 27:51–4. doi: 10.4103/0970-9371.70737 PMC3001175 21157549

[39] Matreja Sandeep S Malukani K Nandedkar Shirish S Varma Amit V Saxena A Ajmera A. Comparison of efficacy of cell block versus conventional smear study in exudative fluids. Nigerian Postgraduate Med J. (2017) 24:245–9. doi: 10.4103/npmj.npmj_150_17 29355165

[40] Theerada A Viboon B Pimpin I. A comparative study of conventional cytology and cell block method in the diagnosis of pleural effuse. J Thorac Dis. (2017) 9:3161–7. doi: 10.21037/jtd.20 PMC5708377 29221292

[41] Jambhulkar M Bhatia JK Singh SK. Correlation between fine-needle aspiration cytology, cell block cytology, and histopathology in the diagnosis of thyroid lesions. J Cytol. (2022) 39:91–7. doi: 10.4103/joc.joc_80_21 PMC9585813 36277809

[42] Mitteldorf C Vilela RS Barros AC Coudry RA. Cell block specimens applied to diagnostic routine of thyroid fine-needle aspiration biopsy. J Bras Patol. Med Lab. (2018) 54. doi: 10.5935/1676-2444.20180009. CYTOPATHOLOGY, ORIGINAL ARTICLE.

[43] Elif K Ferhat O Bahar M. Comparison of liquid-based cytology and cell blocks prepared from cell remnants for diagnosis of cervical pathology. Ann Diagn Pathol. (2024) 69:152265. doi: 10.1016/j.anndiagpath.2024.152265 38266543

[44] Zhang H Wen J Xu PL Chen R Yang X Zhou LE. Role of liquid-based cytology and cell block in the diagnosis of endometrial lesions. Chin Med J (Engl). (2016) 129:1459–63. doi: 10.4103/0366-6999.183431 PMC4910370 27270542

[45] Shi Y Chiaffarano J Yee-Chang M Brandler TC Elgert P Leung A. Self-clotting method improves cell block preparation. Cancer Cytopathol. (2018) 126:190–9. doi: 10.1002/cncy.21950 29178587

[46] Choi SJ Choi YI Kim L Park IS Han JY Kim JM. Preparation of compact agarose cell blocks from the residues of liquid-based cytology samples. Korean J Pathol. (2014) 48:351–60. doi: 10.4132/KoreanJPathol.2014.48.5.351 PMC4215960 25366070

[47] Liu K Dodge R Glasgow BJ Layfield LJ. Fine-needle aspiration: comparison of smear, cytospin, and cell block preparations in diagnostic and cost effectiveness. Diagn Cytopathol. (1998) 19:70–4. doi: 10.1002/(ISSN)1097-0339 9664189

[48] Miceli R Scognamiglio G Camerlingo R Rea G Lettiero A Cantile M. New cell block containing agarose for cytopathological diagnosis of tumor samples. Diagn Cytopathol. (2017) 45:1057–60. doi: 10.1002/dc.23778 28664554

[49] Crescenzi A Baloch Z. Immunohistochemistry in the pathologic diagnosis and management of thyroid neoplasms. Front Endocrinol (Lausanne). (2023) 14:1198099. doi: 10.3389/fendo.2023.1198099 37324272 PMC10266214

[50] Vielh P Balogh Z Suciu V Richon C Job B Meurice G. DNA FISH diagnostic assay on cytological samples of thyroid follicular neoplasms. Cancers (Basel). (2020) 12:2529. doi: 10.3390/cancers12092529 32899953 PMC7564487

[51] Scarpino S Taccogna S Pepe G Papini E D'Angelo M Cascone F. Morphological and molecular assessment in thyroid cytology using cell-capturing scaffolds. Horm Metab Res. (2020). doi: 10.1055/a-1157-6419 32392586

[52] Verri M Scarpino S Naciu AM Lopez G Tabacco G Taffon C. Real-time evaluation of thyroid cytology using new digital microscopy allows for sample adequacy assessment, morphological classification, and supports molecular analysis. Cancers (Basel). (2023) 15:4215. doi: 10.3390/cancers15174215 37686491 PMC10486817

[53] Pausawasdi N Hongsrisuwan P Chalermwai WV Butt AS Maipang K Charatchareonwitthaya P. The diagnostic performance of combined conventional cytology with smears and cell block preparation obtained from endoscopic ultrasound-guided fine needle aspiration for intra-abdominal mass lesions. PLoS One. (2022) 17:e0263982. doi: 10.1371/journal.pone.0263982 35320282 PMC8942242

[54] Rubin R Strayer D Rubin E McDonald J. Rubin's pathology: clinicopathologic foundations of medicine. Philadelphia: Wolters Kluwer Health/Lippincott Williams & Wilkins (2007).

